# Computational analyses identify addiction help-seeking behaviors on the social networking website Reddit: Insights into online social interactions and addiction support communities

**DOI:** 10.1371/journal.pdig.0000143

**Published:** 2022-11-09

**Authors:** Danny Valdez, Megan S. Patterson

**Affiliations:** 1 Department of Applied Health Science, Indiana University School of Public Health, Bloomington Indiana, United States of America; 2 Department of Health Behavior, Texas A&M University School of Public Health, College Station Texas, United States of America; Duke-NUS Medical School, SINGAPORE

## Abstract

**Introduction:**

Although social connection to others with lived addiction experiences is a strong predictor of long-term recovery from substance use disorders (SUD), the COVID-19 pandemic greatly altered global abilities to physically connect with other people. Evidence suggests online forums for people with SUD may serve as a sufficient proxy for social connection, however efficacy of online spaces as addiction treatment adjuncts remains empirically understudied.

**Purpose:**

The purpose of this study is to analyze a collection of Reddit posts germane to addiction and recovery collected between March-August 2022.

**Methods:**

We collected (n = 9,066) Reddit posts (1) r/addiction; (2) r/DecidingToBeBetter, (3) r/SelfImprovement, (4) r/OpitatesRecovery, (5) r/StopSpeeding, (6) r/RedditorsInRecovery, and (7) r/StopSmoking subreddits. We applied several classes of natural language processing (NLP) methods to analyze and visualize our data including term frequency inverse document frequency (TF-IDF) calculations, k-means clustering, and principal components analysis (PCA). We also applied a Valence Aware Dictional and sEntiment [sic] Reasoner (VADER) sentiment analysis to determine affect in our data.

**Results:**

Our analyses revealed three distinct clusters: (1) Personal addiction struggle, or sharing one’s recovery journey (n = 2,520), (2) Giving advice, or offering counseling based on first-hand experiences (n = 3,885), and (3) Seeking advice, or asking for support or advice related to addiction (n = 2,661).

**Discussion & conclusion:**

Addiction, SUD, and recovery dialogue on Reddit is exceedingly robust. Much of the content mirrors tenets for established addiction-recovery programs, which suggests Reddit, and other social networking websites, may serve as efficient tools to promote social connection among people with SUD.

## Author summary

Online forums dedicated to promoting recovery and substance use abstinence are increasingly relied upon by people to build social support systems for recovery. Several studies point to short, medium, and long-term success of these forums at both mitigating substance use while connecting people with shared lived experiences. Our study attempts to corroborate these findings by using data mining to analyze a large collection of posts collected from subreddits specific to addiction and recovery. Using several computer science and automation tools, we analyzed over five-thousand Reddit posts and identified several themes indicative of social support and shared lived experiences including, sharing narratives about addiction struggles, showcasing support for people dealing with addiction, and offering external resources to support someone’s recovery journey. Our findings indeed corroborate literature on this topic which suggests digital support services are sufficient proxies for in-person treatment when such options are unavailable. And, given myriad barriers that may impede people from seeking in-person treatment, such as financial, geographic, and others, our findings also support the broad use of these tools to promote recovery-related support.

## Background

Only 20% of the 40.3 million people in the US aged 12 and older who meet DSM-5 diagnostic criteria for substance use disorders (SUD)—defined as recurrent use of alcohol and other drugs that impedes social function (Substance Abuse and Mental Health Services Administration[1],—receive necessary care [2,3]. Even with treatment, SUD relapse rates range from 40–60% depending on substance class and addiction severity [4]. Regardless of treatment, the single most important predictor of long-term recovery for SUD is social connection to others with lived addiction experiences [5,6].

Social support is cited for its critical role in long-term recovery from SUDs [7–9]. Indeed, a growing body of contemporary and historical studies suggests social networks rooted in aiding recovery are associated with short and long-term substance use abstinence [10]. For example, it was found that after six weeks in an in-patient hospital treatment program, people recovering from alcohol use disorders whose outside social networks supported sobriety showed improvements in substance use and cognitive impairments over and above those whose networks supported alcohol consumption. Similarly, McCutcheon et al [11] found when a person recovering from SUDs’ social network was collectively supportive of their recovery, they were more likely to control urges to use substances, and Patterson et al [12] demonstrated that being more centrally positioned within a recovery program (i.e., having more social connections to other members of the network) was related to longer lengths of sobriety.

Natural experiments, including the COVID-19 pandemic, have reinforced the importance of social networks in aiding addiction and recovery [13,14]. Indeed, since the pandemic was declared a national emergency in February 2020, the Centers for Disease Control and Prevention (CDC) identified a 13% increase in licit and illicit substance use, abuse, and overdose compared with points prior to the pandemic [15]. While anxiety and uncertainty about COVID-19 may have significantly predicted and encouraged substance use during initial stages of the pandemic [16], changes in how people communicate and socialize with one another may equally explain continued increases in substance use rates and decreases in help-seeking behaviors over time [17,18]. For example, January to May 2020 was partially defined by state and local quarantine mandates that barred in-person social interaction. Numerous studies reported how increased social isolation driven by the pandemic also led to increases in internalizing disorder and SUD symptoms [19]. However, as social quarantine mandates relaxed, pandemic-driven social practices remained, including smaller social gatherings, less-frequent travel, increased use of video conferencing software for professional and leisure purposes, and higher observed substance use rates across the lifespan [20]. Due to such communication changes, people living with SUD may be seeking online communities such as blogs or chatrooms as a proxy for in-person treatment at increased capacities [21], though more research on this area is needed [22].

Online forums for addiction and recovery have existed for at least a decade; and been extensively studied as SUD and treatment and recovery adjuncts for myriad substances prior to, and post, COVID-19 [22–24]. Importantly, findings from such studies on Reddit and other social networking tools, as a specific category of SUD treatment, strongly suggest digital networks may be as effective as in-person networks in promoting long-term SUD recovery across a variety of substances and behaviors. Yet, central limitations of these studies remain—including limited sample sizes (of analyzed social media posts) and highly selective inclusion criteria. To our knowledge few studies have empirically studied these spaces with data mining tools intended to consolidate and interpret data en-masse [25]. Advances in natural language processing (NLP) [26] and social network analysis (SNA) [27,28] have made it possible to scrape, model, and visualize online data at unprecedented scope and scale, providing an avenue by which to study online forums germane to addiction and recovery. Interest in data derived from social media is of interest given that, unlike polls and surveys, these data are free response [29], open-ended [30], diachronic [31], and offer insight into digital communication as a proxy for in-person interactions. These unique sides of social media data can lead to robust insights into content for a specific query (i.e., addiction and recovery), psycho-social wellbeing [32], and degree of connection to others [33].

### Present study

This study analyzes a collection of Reddit posts related to substance use addiction and recovery. Our aim with this work is to demonstrate that, in a post COVID-19 world, online spaces and communities may be a suitable venue to connect people with similar lived experiences and promote accountability in the absence of, or in conjunction to, in-person treatment options. Our study is grounded by two research questions:

**RQ1: What themes emerge from a corpus of Reddit social media posts about addiction and recovery?**
**RQ2: How do emergent themes triangulate ongoing research on the efficacy of digital platforms for substance use/abuse support?**

Findings from this study will contextualize how people with SUD communicate in online spaces designed to facilitate social connection with other people struggling with addiction. These findings will also corroborate findings from previous studies on social media as a promoter of substance use recovery, especially those for whom respective social networks are limited.

## Methods

### Data

Data for this study was collected from the social networking website Reddit (January-August 2022). Reddit is an open network of communities where users can engage and connect with others over shared interests, hobbies, or individual experiences. Unlike other popular social media websites used for computational analyses, including Twitter, Reddit is unique in that users can create specific channels to form communities with other interested parties on diverse issues or topics. These channels, otherwise known as subreddits, are thusly comprised of people with shared identities who find, subscribe to, and post within these channels.

In accordance with our research questions, we leveraged Reddit’s Application Programming Interface (API) to identify subreddits germane to addiction. Beginning first with r/addiction—the largest subreddit dedicated to discussing and connecting people with addictive disorders—we queried the API to allocate similar subreddits also spanning addiction related topics. This query returned two more subreddits: r/SelfIimprovement; r/DecidingToBeBetter, which we included for analysis. As these three subreddits do not represent the entirety of Reddit discourse on substance use and abuse, we thusly included additional subreddits specific to addiction and recovery guided by the following inclusion criteria: (1) the subreddit must have been active (i.e., the forum had not been previously archived, truncated, or closed); and (2) the subreddit must have greater than 1,000 active subscribers to ensure our data were robust.

Once we identified our subreddits of interest based on our inclusion criteria, we queried the API to collect new posts and top posts from (1) r/Addiction; (2) r/DecidingToBeBetter, (3) r/SelfImprovement, (4) r/OpitatesRecovery, (5) r/StopSpeeding, (6) r/RedditorsInRecovery, and (7) r/StopSmoking. Upon filtering our data for duplicates our final sample size comprised (n = 9,066) posts. Though this study of anonymized secondary data was exempt from IRB review, our use of these data strictly adhered to the Institutional Review Board’s standards for secondary data collection and ethical social media mining (for more insight into ethical considerations for social media mining and analysis see [34]).

### Analyses

We leveraged several unsupervised natural language processing (NLP) analyses: (1) term frequency invariance document frequency (TF-IDF); (2) k-means clustering, and (3) principal components analysis (PCA). We also ran a Valence Aware Dictionary and sEntiment [sic] Reasoner (VADER) sentiment analysis to calculate affect.

### Term Frequency Invariance Document Frequency (TF-IDF)

TF-IDF is the process of assigning weights, or degree of importance, to every word in a corpus [35–37]. To create weights, the TF-IDF algorithm calculates two values: (1) the total number of times a word is mentioned in a document (i.e., Reddit post); contrasted with (2) the number of times the same word is mentioned across all other documents (i.e., all other Reddit posts). This analysis creates weights and vectors for each term (and larger document) which are then compared and grouped by a computer using K-means clustering.

### K-Means clustering

K-means clustering, a powerful and highly efficient machine learning tool, takes data that have been assigned weights through TF-IDF and sorts them into one of *k* clusters [38–40]. These clusters represent a theme, of sorts, that group posts together by content and semantic similarity guided by TF-IDF vectors. To identify the optimal number of clusters for a corpus, we rely on the sums of squared differences for a range of possible clusters. Scree plots generated by the K-means analysis revealed a two or three cluster solution.

### Principal Components Analysis (PCA)

Matrices produced by the TF-IDF and k-means clustering algorithms are highly complex, multidimensional, and difficult to interpret [41]. To simplify these matrices for data visualization purposes, we apply a PCA Analysis, which reduces the data into two dimensions—a common setting for data visualization in NLP analyses. A two-dimensions setting for a principal components analysis allows data to be visualized in two dimensions. A three-dimension setting would yield a three-dimensional figure [41]. These coordinates are then used to plot and visualize the data along dimensions outlined from the K-means analysis. To determine model fit, we applied a cross-validation logistic regression for a two and three k-means cluster solution, which is explained at length in the procedure section of this paper.

### VADER sentiment analysis

VADER sentiment analysis refers to an algorithm and analysis that examines the polarity of words within each social media post. Posts are fed through a lexicon, or online dictionary, that is pre-coded with values for all positive and negative words in the English language. Negative VADER values denote lower affect (i.e. -.99 through -.01); positive values denote higher affect (i.e., .01 through .99) [42]. Here, positive affect denotes the presence of positive words (therefore a positive or supportive posts); negative affect denotes the presence of negative words (therefore a negative or somber post).

### Procedure

**Fig 1:**
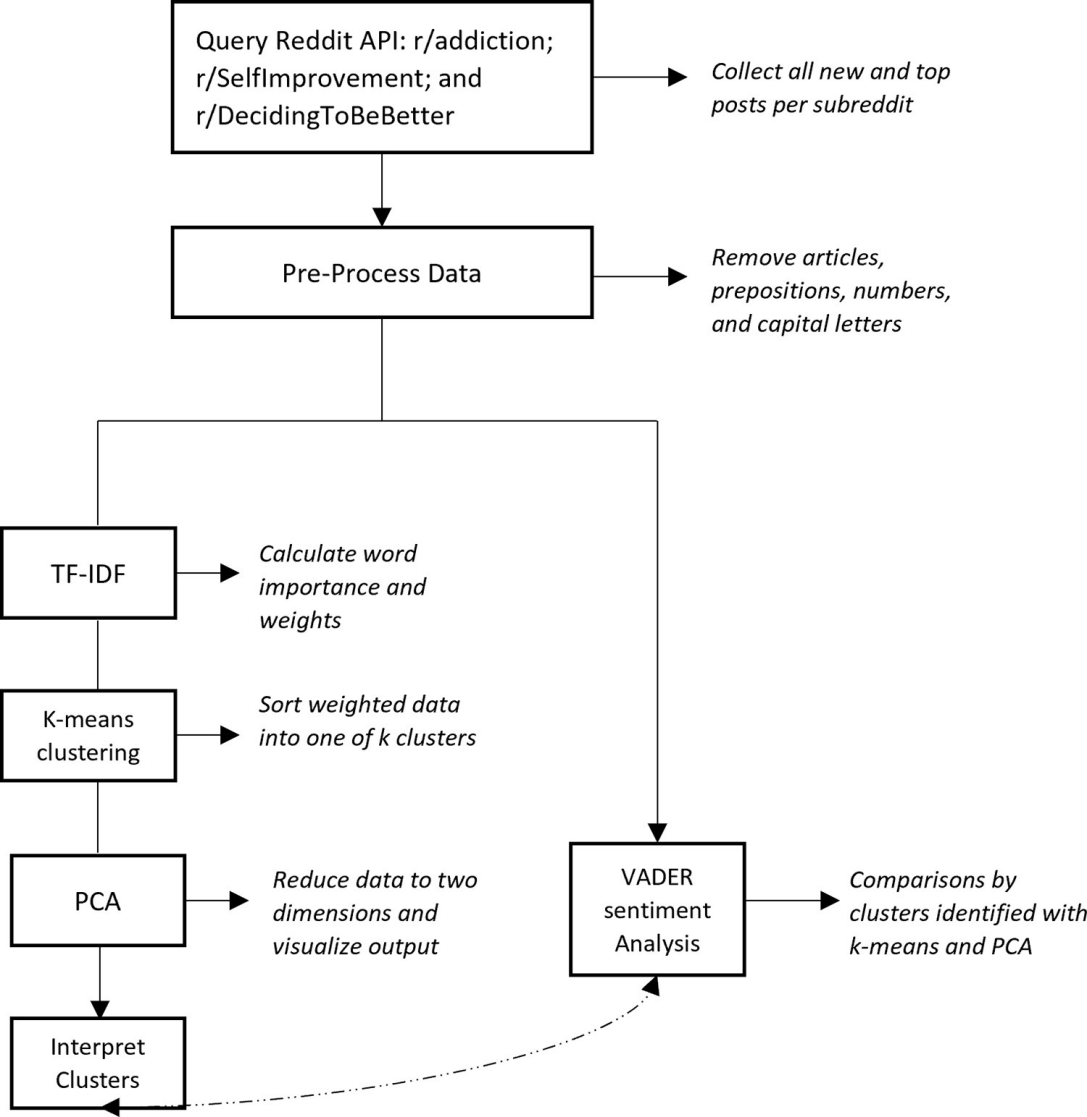
Workflow detailing data cleaning analysis procedures.

Our workflow is depicted in Fig 1. First, we queried the Reddit API to archive top and new posts from the addiction subreddits included for analysis. After removing duplicate and non-English posts, we pre-processed our data to remove parts of speech that would detract from the clarity of our models, including articles, prepositions, punctuation, abbreviations, and numbers. Once our data were pre-processed, we ran a TF-IDF to generate weights for our words. We then ran a K-means clustering analysis to identify the optimal number of topics for our corpus. The sums of squared differences with elbow scree plot projections revealed a two or three-cluster solution. We then programmed our PCA to construct a two-cluster model and a three-cluster model across two dimensions and visualized our data on a vector map.

To check for the accuracy of our data classification, we then performed a k-fold cross-validation with logistic regression—a process where a computer subsets data and then predicts the likelihood the subset data can be re-sorted into its original class. Our logistic regression cross-validation yielded 71% accuracy for a two-cluster solution, and 85% accuracy for a three-cluster solution, indicating a more accurate model fit for the three-cluster solution. As such, our remaining analyses and interpretation of outcomes occurred using the three-cluster model. We lastly ran a sentiment analysis for each post across the three clusters.

## Results

Our findings reveal the extent that people struggling with addiction, broadly defined, use Reddit to connect with others sharing similar lived experiences. We present our findings briefly below without comment.

### Principal component analysis: 3-Cluster solution

**Fig 2:**
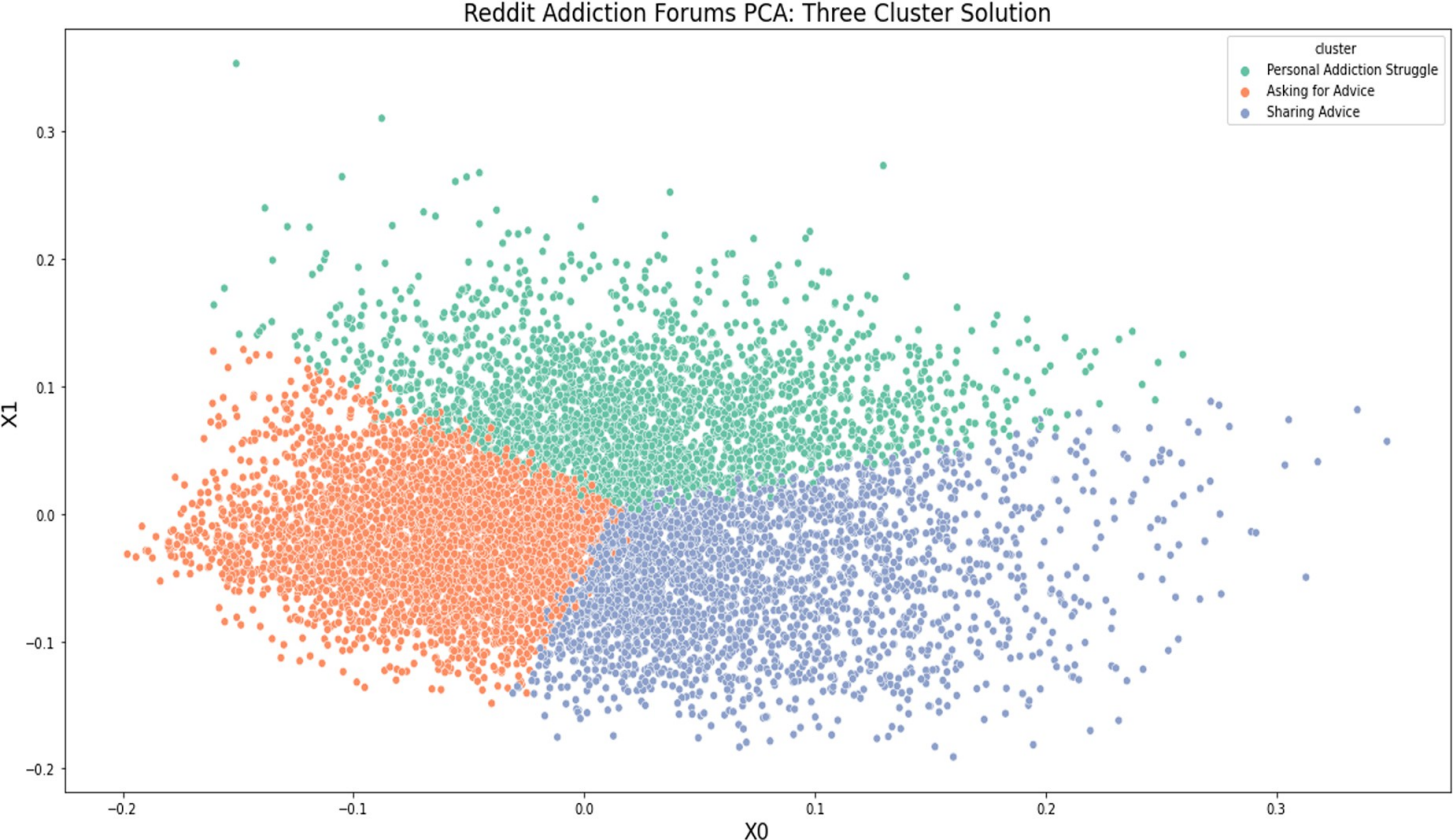
The following vector map reflects a k-means clustering and principal components analysis (PCA) for 9,066 Reddit posts collected from addiction and recovery subreddits.

Results from our PCA indicated our data fit a three-cluster solution (see Fig 2). To determine what each cluster represented, we reviewed 50 randomly selected posts to ascertain general and specific themes. Table 1 provides a list of excerpts from posts pulled within each cluster, which were abridged and anonymized to prevent identification. After reading excerpts and upon unanimous agreement across two reviewers, we named each centroid accordingly: (1) Asking for advice: Sincere solicitations of advice and insight from people with shared lived experiences, (2) Giving advice: People offering advice and insight about their lived experience with addiction, and (3) Personal addiction struggle: Sharing narrative accounts of personal battles with addiction.

**Table 1:** Example abridged and anonymized Reddit posts pulled from each centroid outlined in the PCA vector map.

Asking for advice	What is your best tip for getting over cravings and possible relapse?
	What do you do to show yourself some love on particular rough days, that isn’t self-destructive?
	Any suggestions on how I can finish my degree while simultaneously improving my life?
	4 months clean from ice…. How quickly did things turn around for you within 2 years?
	What is the best way to get off fentanyl? Does Suboxone work at all? If I were to use kratom, how would I transition being I just used Suboxone? Thank you for your time. This is my first post. I am usually searching for answers.
	No one is prouder for being one month sober than me—but I have a question—does it ever get any easier.
	How do I talk to my [redacted] on what I suspect is an opiate relapse? He’s withdrawn lately, and I am really worried.
Giving Advice	I need to start living again, because right now I feel like every one of my actions is directed towards the next high. If I can do it you can too!
	Don’t beat yourself up. You’ve got this.
	So just wanted to say that if you feel like you did nothing in the lockdown then that’s okay because you did give yourself a rest, you gave yourself time to reflect on yourself and that is as valuable as doing other "productive" things as well!
	I just wanted to give some words of advice/counsel to people who are at the younger end and still have some time to change. Just push it and push it because you will never get this time back.
	I feel you. I’ve been there when I had nothing, was depressed, and didn’t know what to do. I just struggled and struggled until one day I realized I have my shit together pretty good.
	Hey, you have to remember to just take it one day at a time. Some days are easy; but some will be unbearable. You will mess up, and that’s okay and part of the process
	Take it from someone who had dual addiction to alcohol and fentanyl—you can get through this. Just take it slow. Get help. Reach out. You are not alone. I got clean on this subreddit you can too
Personal Addiction Struggle	Tonight is my first night without Oxy/Fentanyl. I’m so scared. Luckily, my mom is with me. I have to stay clean for the next 45 hours before I can take my Suboxone.
	For the first time I woke up determined I wasn’t going to use. No questions. For the first time in a year, I feel like I can move forward. My life can rebuild. F*ck you heroin. I’m over you.
	Today, I don’t rely on substances to get me through life. I’m in the healthiest relationship of my life, I’m going back to school to become a nurse and all of my relationships that I ruined are being fixed. Today I think about the boyfriend I had back then, who passed from an overdose last June at the age of 25.
	The last time I relapsed I was hospitalized. It had been 11 months before that time so I thought I was doing well and wouldn’t do it again. I was wrong. Maybe from that experience I have been more compelled to stop. Idk. Either way I’m proud of myself for coming this far.
	So I would of had 3 years off of opiates this November but ended up having a horrific overdose Tuesday. Laying in my hospital bed now just waiting for the trazadone to kick in.
	I’m trying to not smoke dope, but snorting it doesn’t really do anything for me, and it feels like dope is the only thing that slightly subsides the pain of this. I’m back to being dope sick again, my tolerance is high and I’m spending all of my money on this sh*t.
	I decided I’m quitting yesterday and it’s the first time I’m having the intention to do so, have taken breaks and it wasn’t really the same. It’s a mixture of resentment and clarity. I was a afraid because I didn’t want to try, because I could just not keep the obviously self-deceptive ideas that "one day.."

Descriptive statistics for each centroid indicate that 44% of collected posts (n = 3,885) correspond to the ‘Asking for advice’ cluster; 31% (n = 2,661) correspond with the ‘Giving Advice’ cluster; and 25% (n = 2,159) correspond with the ‘Personal Addiction Struggle’ cluster.

In addition to determining content within each cluster, we also programmed a macro to identify a list of all addictions possibly mentioned in our dataset. This search revealed that a vast majority of posts pertained to use and abuse of licit and illicit substances (e.g., alcohol, tobacco, cocaine, heroin, and others). However, pornography—as a non-substance driven addiction—was frequently mentioned as well, usually in the context of a co-occurring addictions (e.g., opiates and pornography addiction). Table 2 outlines the most commonly identified addictions mentioned in the dataset. To determine affect within each cluster we used a VADER sentiment analysis, Table 3 shows the means and standard deviations for VADER scores across the three centroids. The ‘Giving advice’ cluster had the highest observed affect. By contrast, ‘Personal addiction struggle’ had the lowest observed affect.

**Table 2:** Top addiction disclosures on Reddit and frequency of mentions.

Addiction	Frequency
**Marijuana**	1032
**Heroin**	978
**Alcohol**	802
**Cocaine**	419
**Pornography**	115
**Crack**	176
**Fentanyl**	134
**Ecstasy**	59

**Table 3:** VADER sentiment analysis per cluster.

		Asking for Advice	Sharing Advice	Personal Addiction Struggle
VADER	Mean	0.29	0.44	0.18
	SD	0.68	0.79	0.78

A one-way ANOVA comparing mean affect per cluster was statistically significant F(2, 5082) = 31.94, p < .001. Tukey’s post-hoc contrast revealed the mean VADER score per cluster was significantly different for all contrasts except for Asking for Advice and Sharing Advice, which was not statistically significant (p = .026).

## Discussion

Findings from this study revealed diverse ways in which addiction and substance use dependence are discussed on subreddits dedicated to addiction and recovery. Broadly, our findings align with previous findings that suggest digital forums are a sufficient proxy treatment.

### NLP analysis of Reddit data corroborates research in online forums for social connection

Our study contributes to ongoing research establishing the benefit and efficacy of social media and similar digital platforms as social connection tools to aid in addiction and recovery. In alignment with previous research, our findings strongly suggest that people are willing to disclose personal struggles with addiction and share words of advice for others who are seeking treatment and/or connection through digital mediums. Indeed, this larger body of work frames Reddit (and similar digital tools for addiction and recovery journeys) as sufficient proxies for in-person therapy sessions, with our study contributing a layer of validation to those findings as they relate to how these platforms are utilized by others.

Within our data, “Asking for advice” emerged as the primary theme from Reddit data, suggesting social media could serve as a place for support and information-seeking for people struggling with addiction. Previous research has established the importance of social support for people recovering from SUD [9,13,43], and advice seeking is one way to receive informational, emotional, and tangible support from others. The second most common theme from our analysis was “giving advice” which we contextualized as referring to acts of service. “Service” is commonly the final step within 12-step recovery programs [44], where people who have successfully navigated their recovery journey help others to do the same. Studies on recovery communities find that people feel a responsibility to return the support provided to them as they navigated recovery [45]. Willingness to share advice or struggles does not seem to be specific to one or a select group of addictions—rather, we identified a multitude of substances mentioned within our data, including those specific to a reddit forum (i.e., opiates and alcohol) and other substance addictions that co-occurred with other addictions (i.e., ecstasy and crack cocaine). Finally, “personal addiction struggle” was the final theme that emerged from Reddit data. In addition to service, a common practice in recovery groups is to share stories and lived experiences [46]. Because this was an addiction-focused online group, and assumingly a community of people dealing in some way with addiction, sharing personal narrative is commonplace; and given the popularity of these forums it is likely any single person can find a person (or groups of people) whose journey mirrors their own. Indeed, storytelling and story sharing is an important aspect of personal healing [47], and can be a resource to others navigating a similar path.

Even with numerous cost-effective in-person or residential treatment options for managing SUD, only one in five people who meet criteria for a SUD receive treatment. The primary reason people may not seek care is due to the perception drug and alcohol use may not be a problem and thusly will forgo or deny treatment when offered [48]. Additionally, addiction and SUDs are highly stigmatizing disorders [49]. As such, people may not feel comfortable in in-person treatment programs or recovery meetings due to the perceived stigmatization of seeking help from friends, families, or group members. Beyond stigmatization and inability to perceive a problem with substance use, many are limited by access to residential treatment programs, especially in remote locations or areas with high poverty [50]. This is an ongoing challenge in residential treatment, namely, to offer alternatives that are cost-effective and accessible [51]. Our findings suggest online communities and spaces such as Reddit could be an important primer for future treatment. If people are using a less-clinical space for advice, it could be their first step in recognizing a problem in themselves or in people to whom they are closely connected [52]. The autonomy offered through social media, coupled with layers of anonymity provided through platforms like Reddit [53,54], could be giving people a safe space to learn more about addiction and potentially get help based on response from others within the addiction community [55]. Reddit (and other social networking websites) are also free to use, which implies that people who may not have access to treatment could, at the very least, access digital forums for support and resources.

### Social media as a viable online tool for outreach and improved network connection

Our findings equally illustrate that social media is a viable and user-friendly tool for people to connect with others with shared experiences in addiction and recovery. This observation accompanies important insights into theoretical network composition and in-group cohesion for online forums related to addiction. First, our Reddit data scrape included a representative sample of forums dedicated to substance use addiction. While we expected channels to discuss various facets of broad substance use addiction, we were surprised at the specificity at forums dedicated to specific drugs and substances, including alcohol, tobacco, opiates, and methamphetamines. Our findings also revealed that content embedded within these channels equally suggested co-occurring SUDs. Yet, regardless of the substance, or subreddit analyzed herein, all content generally adhered to the same style of discourse related to help seeking, advice sharing, and narrative sharing for substance use related addictions. This suggests user-posted content on these forums is welcoming to people struggling with any broad range of substance addiction; and these subreddits *might* be principally used by people who are currently battling addictions related to overreliance on external substances.

Extensive research documents that perceiving improvement in one’s social network can improve subjective wellbeing and other physical health outcomes [13,56]. Therefore, identifying and associating with forums that intend to connect people with shared substance addictions may facilitate such perceived improvements in one’s personal social network. Because subreddits germane to addiction are seemingly comprised of people who, at some capacity, can relate to substance use addictions, then Reddit may be a platform to promote social networks among people with shared lived experiences. These shared experiences may foster a sense of community, which may be especially beneficial for people with SUD who could not otherwise attend in-person groups or afford in-patient treatments [57]. And, with over a dozen subreddits specific to addiction, we conclude a single social media user can find a community to assist in personal and collective recovery journeys. People can also identify resources specific to their own type of addiction that is free and immediately accessible.

### Implications and recommendations for using online spaces to promote SUD related interventions

This study affirms the importance of social media as community support in the addiction process and justifies continued efforts to foster support networks for those pursuing recovery from SUD. Yet, despite strong evidence that social media may represent a viable source for social connection among people with SUD, the tenuous and often negative aspects of social media must also be addressed. In this vein, interdisciplinary research continuously highlights the negative mental health effects of chronic social media use [58]. Examples of poor outcomes of chronic social media use include worsening of internalizing disorder symptoms [32], perceptions that friends are happier than the user (happiness paradox; [27], and disruptions in sleep wake cycles [59,60]. Though more research is needed on social media’s physical and mental health effects on people with SUD, it is likely that constantly viewing social media feeds and overreliance of social media for social connection may have negative outcomes within this population, which could include diminished effectiveness in promoting long term recovery.

Through our study, we have showcased positive aspects of social media for SUD recovery through narrative sharing and help-seeking behaviors. However, we caution future researchers and interventionists from relying on social media as a unilateral source of SUD treatment and intervention. Social media can, and should, be used by people with SUD on their own accord to connect with others through online forums. However, regulating people’s social media feeds as part of an intervention or mandating people with SUD to use more social media may have negative consequences such as those outlined above. To mitigate unintended consequences and affirm social media’s role in 21st century treatment for SUD, we offer two practical recommendations, including leveraging social media as a tool to identify populations of interest for mobile health, third party interventions, and learning latent psychopathology behind scraped social media feeds.

The majority of social media platforms offer application programming interfaces (APIs) that allow developers and researchers to scrape social media data along specific queries. This implies that on Twitter, for example, we can identify populations of interest based entirely on a query of select words or phrases. The phrase “I am struggling with addiction” or “I am addicted to” would serve as initial queries to find US adults who are posting unique social media posts containing this phrase or groups of phrases. Assuming these adults are genuine about such disclosures (empirical research suggests they are [30]), automated bots can be used to contact these people and triage them into digital or mobile health third party campaigns not affiliated with a social media platform [61]. Similarly, timelines from users posting about their recovery journey can also be leveraged to create classifiers—otherwise known as trained algorithms that learn writing and communication patterns from a representative sample [62]—that can predict the degree to which someone on social media may have or be susceptible to a SUD. Both recommendations would aim to improve sampling among people with SUD and serve as preliminary data to improve long-term effectiveness of other interventions. However, we acknowledge that more research on classifiers as intervention tools is needed, particularly as they relate to the ethics of creating a digital ‘voice’ for SUD derived from people’s personal, online disclosures of their struggles with addiction and SUD. Indeed, these vulnerable populations are susceptible to exploitation, particularly regarding information leaks that may inadvertently ‘out’ a person with an SUD without their approval or consent.

### Limitations

This study is subject to limitations we hope to address in future work. First, the anonymity of social media data makes it difficult to determine the authenticity or genuine nature of Reddit posts. Thus, while we should assume these posts are genuine and derive from people with SUD, we must be cautious in making definitive claims about our data and findings therein. A second limitation involves our reliance on NLP algorithms to sort and classify our data. Though these algorithms are known for being exceedingly precise and attuned to social media vernacular, computers and similar machines cannot make definitive judgements about content as effectively as humans. Additionally, the algorithms used for this study were entirely unsupervised, meaning our data was run against a program that had not been pre-trained on human language. Therefore, it is likely some Reddit posts, may have been misclassified by the k-means and PCA analyses. We also acknowledge that we did not perform a full qualitative analysis with these data, and only reviewed between 50–75 posts per cluster to ascertain meaning. This naturally implies that there are likely very niche discussions embedded within our data. Given that these data are publicly available we encourage others to perform a more robust qualitative analysis in the future. To address these limitations, we propose additional studies with these data (and other Reddit data more broadly). Specifically, future studies should consider an analysis of Reddit data using supervised machine-learning algorithms trained on human language. These algorithms may reveal more robust topics embedded within each cluster.

## Conclusion

Social support networks are widely considered the strongest predictor of long-term SUD recovery. However, changes in personal and group communication habits, partially stemming from the COVID-19 pandemic, have brought new attention to the role and efficacy of online social support groups as a proxy for in-person interactions. Our findings illustrate that people use Reddit to disclose addiction struggles and seek support from others. Though much work remains to examine how effective Reddit, and other online support systems, are at mitigating SUD and promoting recovery, our cursory evidence suggests social media continues to play a pivotal role in promoting connection regardless of location, income, or access to formal treatment centers.
